# Neoadjuvant concurrent chemoradiotherapy with and without hyperthermia in retroperitoneal sarcomas: feasibility, efficacy, toxicity, and long-term outcome

**DOI:** 10.1007/s00066-021-01830-0

**Published:** 2021-11-04

**Authors:** A. Willner, K. Fechner, A. Agaimy, F. Haller, M. Eckstein, O. J. Ott, F. Putz, U. S. Gaipl, S. Kersting, N. Meidenbauer, R. Grützmann, R. Fietkau, S. Semrau

**Affiliations:** 1 Department of Radiation Oncology, University Hospital Erlangen, Friedrich-Alexander-Universität Erlangen-Nürnberg, Universitätsstraße 27, 91054 Erlangen, Germany; 2 Department of Surgery, University Hospital Erlangen, Friedrich-Alexander-Universität Erlangen-Nürnberg (FAU), Maximiliansplatz 1, 91054 Erlangen, Germany; 3 Institute of Pathology, University Hospital Erlangen, Friedrich-Alexander-Universität Erlangen-Nürnberg (FAU), Krankenhausstraße 8–10, 91054 Erlangen, Germany; 4 Department of Haematology and Oncology, University Hospital, Friedrich-Alexander-Universität Erlangen-Nürnberg (FAU), Ulmenweg 18, 91054 Erlangen, Germany

**Keywords:** Thermotherapy, Doxorubicine, Ifosfamide, Liposarcoma, Leiomyosarcoma

## Abstract

**Purpose:**

Retroperitoneal (RPS) sarcomas are associated with poor local and abdominal tumor control. However, the benefit of preoperative radio- or chemotherapy alone for these entities is currently unclear. Moreover, as intermediate- and high-grade sarcomas have a tendency toward early metastasis, exploration of neoadjuvant strategies is of high importance. This analysis reports the results of our 20-year single-institution experience with preoperative neoadjuvant concurrent chemoradiation.

**Methods:**

From 2000–2019, 27 patients with intermediate- or high-grade RPS (12 dedifferentiated liposarcoma, 10 leiomyosarcoma, 5 others) were treated with radiotherapy (median dose: 50.4 Gy; range 45–75 Gy) and two cycles of chemotherapy (doxorubicin 50 mg/m^2^ BSA/d3 q28 and ifosfamide 1.5 g/m^2^ BSA/d1‑5 q28) in neoadjuvant intent. Chemotherapy consisted of doxorubicin alone in two cases and ifosfamide alone in one case. Fifteen patients (56%) additionally received deep regional hyperthermia.

**Results:**

The median follow-up time was 53 months (±56.7 months). 92% of patients received two cycles of chemotherapy as planned and 92% underwent surgery. At 5 and 10 years, abdominal-recurrence-free survival was 74.6% (±10.1%) and 66.3% (±11.9%), distant metastasis-free survival was 67.2% (±9.7%) and 59.7% (±11.1%), and overall survival was 60.3% (±10.5%) and 60.3% (±10.5%), respectively. CTC grade III and IV toxicities were leukocytopenia (85%), thrombocytopenia (33%), and anemia (11%). There were no treatment-related deaths.

**Conclusion:**

Neoadjuvant chemoradiotherapy with and without hyperthermia for retroperitoneal sarcomas is feasible and provided high local control of intermediate- and high-grade sarcoma.

## Background

Retroperitoneal sarcoma (RPS) are rare and challenging oncologic entities. Liposarcoma and leiomyosarcoma are the main histologic types encountered, with high-grade histology being present in half of all cases [1–3]. Although surgical resection is the mainstay of treatment for cases without distant metastasis, most patients ultimately develop intra-abdominal recurrences after resection alone [4], which is the rationale for additive radiation treatment (RT). Results of retrospective case series suggest that postoperative radiotherapy (PORT) may provide increased local tumor control [5–7]. There are, however, limitations for the implementation of PORT, such as problems in defining the postoperative volume to be irradiated after resection and the risk of higher long-term gastrointestinal toxicity, as abdominal organs settle back into their regular positions after surgery [8]. On the other hand, preoperative radiotherapy has the advantage of a more straightforward definition of the clinical target volume and the reduction of late side effects in patients with soft-tissue sarcoma of the limbs [9]. The STRASS-Trial by the European Organisation for Research and Treatment of Cancer (EORTC) investigated the benefits of preoperative radiotherapy for RPS and showed that neoadjuvant radiotherapy is well tolerated but does not improve abdominal recurrence-free survival (AFRS) [10]. Subgroup analyses revealed that preoperative radiotherapy significantly improved AFRS in low-grade sarcoma and liposarcoma, but not in high- and intermediate-grade sarcoma, suggesting that the different grades of RPS require a differentiated treatment approach according to tumor grade.

These findings raise the important question of whether the addition of chemotherapy (CT) and hyperthermia (HT) to radiotherapy might result in improvement of intra-abdominal control rates. To explore this question, this retrospective analysis was designed to assess the treatment outcome of RPS patients who received combined chemoradiotherapy (CRT) at the Department of Radiation Oncology, University Hospital of Erlangen. The reason for concomitant application was to enable complete surgical resection and lower local recurrence rates.

In recent years, neoadjuvant concurrent chemoradiotherapy (CCRT) has been expanded to additionally include hyperthermia in selected cases [11]. The aim of the concomitant application was to ensure a complete resection and lower the risk of local recurrences.

## Materials and methods

### Patient characteristics

All data on the course of disease and follow-up of all sarcoma cases entered in our hospital database since 2000 were retrospectively evaluated (see Table 1). The cut-off date for analysis was August 31, 2019. The management of each case was discussed primarily in a multidisciplinary team setting before and after treatment, informed consent was obtained from all participants. According to the unicentral established therapy regimen, biopsy followed by neoadjuvant concurrent chemoradiotherapy was performed in all cases with confirmed FNCLCC grade G2 or G3 tumors. Neoadjuvant chemotherapy alone was not performed. Exclusion criteria for neoadjuvant CCRT were ECOG >1, intestinal passage obstruction, and/or age ≥80 years. Patients with a high risk of severe renal failure following radiotherapy with or without subsequent nephrectomy were also excluded. This was determined by MAG3 scans (isotope nephrography) for the individual assessment of left and right renal function. Histological diagnosis was based on the World Health Organization (WHO) classification of soft tissue tumors valid at initial biopsy diagnosis. Tumor grading was performed according to the French grading system (FNCLCC).

**Table 1:** Patient and tumor characteristics

**Patients**	***N*** **=** **27 (100%)**
*Gender*	
Male	14 (52%)
Female	13 (48%)
*Age at first diagnosis*	61 years (range 24–80 years)
*Median follow-up*	53 months
**Tumor**	***N*** **(%)**
*Primary tumor*	18 (67%)
*Recurrent tumor*	9 (33%)
*Histological type*	
Liposarcoma	13 (48%)
Leiomyosarcoma	10 (37%)
Pleomorphic sarcoma	4 (15%)
*Grading*	
G1	1 (4%)
G2	12 (44%)
G3	14 (52%)
*Tumor stage*	
T1	1 (4%)
T2	20 (74%)
T3	2 (7%)
T4	4 (15%)
*Nodal stage*	
N0	27 (100%)
*UICC 2017*	
IB	1 (4%)
II	1 (4%)
IIIA	18 (67%)
IIIB	7 (26%)
*Mean tumor diameter*	122 mm (range 35–350 mm)

### Treatment

#### Radiotherapy

**Fig. 1:**
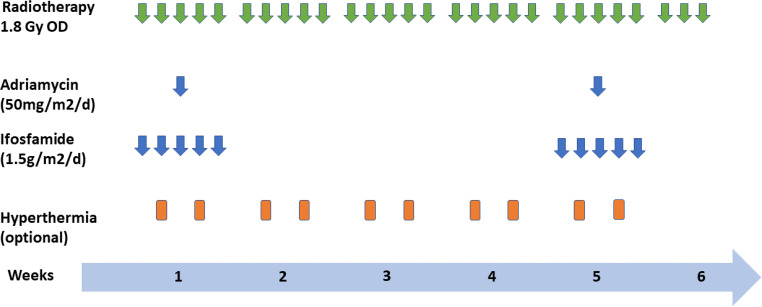
Therapy regimen for neoadjuvant concurrent chemoradiotherapy ± hyperthermia in retroperitoneal sarcomas

The radiation techniques used were multi-field 3D RT with a dose prescribed to the ICRU50 reference point until 2012, and intensity-modulated radiation therapy (IMRT) or volumetric modulated arc therapy (VMAT) in subsequent years. Doses were generally delivered in fractions of 1.8 Gy, five times a week. However, three patients received fractions of 1.5 Gy instead. Four patients treated before 2010 had a treatment protocol specifying a 7-day treatment break in week 3 (see Fig. 1).

#### Chemotherapy

Twenty-four patients received a combination regimen of doxorubicin (50 mg/m^2^/d on day 3) plus ifosfamide (1.5 g/m^2^/d on days 1–5) in weeks 1 and 5 (if they had sufficient cardiac and renal function) with appropriate antiemetic therapy (HT3 antagonist), hydration, and cystitis prophylaxis. Three patients had comorbidities at baseline that required chemotherapy dose reduction. The first cycle of chemotherapy was started in parallel with radiation. Granulocyte colony-stimulating factor (G-CSF) was not administered prophylactically but was given to patients with leukocytopenia (count <1500/µL). To receive the second cycle of chemotherapy in week 5 of radiotherapy, patients had to have a leukocyte count greater than 3000/µL and a platelet count greater than 100,000/µL. If not, the second cycle was postponed by a maximum of 2 weeks or was not performed in case of inadequate restitution of hematopoiesis. Postoperative or consolidating chemotherapy was not performed in patients with inoperable disease.

#### Hyperthermia

From 2004 on, regional hyperthermia was performed with an SD 2000 hyperthermia system (BSD Medical/Pyrexar, Salt Lake City, UT, USA) with or without non-invasive MR thermometry with 1–2 treatments/week. The target temperature was 40–44 °C for 60 min per treatment.

#### Surgical resection

**Fig. 2:**
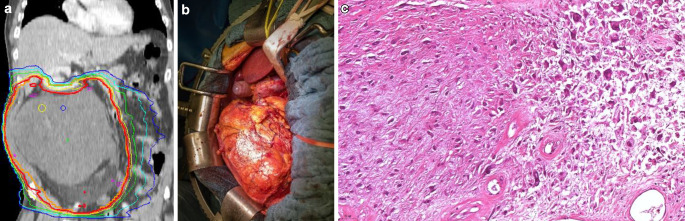
Retroperitoneal liposarcoma with dose distribution of kidney-sparing radiotherapy (**a**), surgical site of en block resection (**b**); strongly regressive altered residual tumor tissue after chemoradiotherapy (**c**)

En bloc resection of the tumor was to be carried out 6–10 weeks after the end of chemoradiotherapy if possible. The treatment regimen is illustrated in Fig. 1. Additional pictures detailing the treatment regimen are shown in Fig. 2.

### Follow-up and statistics

The feasibility of chemotherapy was verified based on data in the patient treatment records and pharmacy records. The performance of radiotherapy was confirmed using the MOSAIQ Record & Verify System. Treatment toxicity was graded as per the Common Terminology Criteria for Adverse Events (CTCAE) version 4.0 [12]. Postoperative complications were assessed according to data in the electronic patient records. After the treatment, patients were scheduled for quarterly clinical and semi-annual computed tomography (CT) follow-up assessments. All patients without reported incidents were contacted by phone on the follow-up assessment date. Two patients were lost to follow-up. The data from their last clinical visits were included in the analysis.

Endpoints were abdominal recurrence-free survival (ARFS, defined as the time to abdominal relapse after R0, R1, or R2 resection), distant metastasis-free survival (DMFS), disease-free survival (DFS), and overall survival (OS).

Statistical analysis was performed using SPSS Statistics Version 26 (IBM Corp., Armonk, NY, USA). Survival analysis was performed by means of the Kaplan–Meier method and the logrank test, as well as Cox regression analysis. Differences of *p* < 0.05 were defined as statistically significant.

## Results

### Patients

Of the 27 patients included in the analysis, three ultimately received definitive treatment because resectability was not achieved: persistent inoperability was confirmed by the multidisciplinary team based on the results of repeat imaging studies after the patients had received 50.4 Gy of radiation. Twenty-four patients were classified as potentially resectable and underwent surgery during the further course. Characteristics of the patient population are summarized in Table 1. Of the 27 patients, 26 (96%) had intermediate- and high-grade sarcomas and one (female) had a single-site, multi-recurrent, low-grade liposarcoma. The histological subtype was dedifferentiated liposarcoma in 12 cases (7 intermediate grade and 5 high grade), leiomyosarcoma in 10 patients (5 intermediate grade and 5 high grade), and 4 other subtypes (4 high grade).

### Treatment feasibility

All patients received the intended prescription dose of 45–55 Gy in the neoadjuvant setting and 60–65 Gy in the definitive setting. The median radiation dose (with standard deviation, SD) was 54.4 ± 4.1 Gy in the neoadjuvant setting and 65.0 ± 8.7 Gy in the definitive setting (see Table 2).

**Table 2:** Treatment specifications

**Radiotherapy**	***N*** **(%)**
*Method*	
Intensity-modulated	8 (30%)
3D	19 (70%)
*Fraction size*	
1.5 Gy	3 (11%)
1.8 Gy	3 (11%)
2.0 Gy	21 (78%)
*Boost*	19 (70%)
*Fractionation in overall dose*	
<50 Gy	1 (4%)
50–54.9 Gy	10 (37%)
55–60 Gy	14 (52%)
>60 Gy	2 (7%)
*Treatment delay*	
<5 d	23 (85%)
≥5 d	4 (15%)
**Chemotherapy**	***N*** **(%)**
*Type of chemotherapy*	
Doxorubicin + ifosfamide	24 (89%)
Ifosfamide only	1 (4%)
Doxorubicin only	2 (7%)
*Dose of chemotherapy*	
<50% of planned dose	7 (26%)
≥50% of planned dose	20 (64%)
*Cycles of chemotherapy*	
<2 cycles	2 (7%)
≥2 cycles	25 (93%)
**Hyperthermia**	***N*** **(%)**
*Hyperthermia*	15 (56%)
<5 treatments	8 (53%)
≥5 treatments	7 (47%)

Concomitant chemotherapy consisting of doxorubicin plus ifosfamide was administered to 24 patients. Of these patients, 7 (29%) received >80% of the planned dose of ifosfamide, while 11 (46%) received >50% and 6 (25%) received 30–50% of the total dose of 15 g/m^2^ of ifosfamide planned for the two cycles. For doxorubicin, 20 (83%) of the patients received more than 80% of the planned dose of 100 mg/m^2^ over two cycles, and the remaining 4 patients received 50 to 80% of the target dose. Chemotherapy was administered according to protocol in 24 cases (89%).

Due to age >80 years, two patients received weekly full-dose doxorubicin monotherapy for at least five cycles (to a total dose of 75 mg/m^2^). In the remaining case, a patient pretreated with doxorubicin received ifosfamide parallel to radiotherapy at a dose of 1.5 g/m^2^/d on days 1–5 but could not receive the second cycle due to severe deterioration of his general health.

Hyperthermia treatment (HT) was administered parallel to RCT in 15 (56%) patients who received a median of five HT treatments.

Of the patients who received neoadjuvant therapy, 16 (67%) were classified as R0 after en bloc resection (see Table 2), 6 (25%) as R1. The resection status of 2 (8%) could not be determined due to missing data at the time of the analysis.

### Toxicity profile

The most common adverse events of chemoradiotherapy were hematological side effects (see Table 3). Surgical resection could be performed at the planned time in all cases. The most common AEs were radiation dermatitis (19%, none CTCAE grade 3 or 4) and diarrhea (6%). Eight patients (26%) experienced nausea throughout the treatment. Postoperative complications were acute renal failure (*n* = 1), burst abdomen (*n* = 1), anastomotic leakage (*n* = 1), and cerebellitis (*n* = 1) 3 weeks after surgery. The case of cerebellitis was determined to be autoimmune and resolved spontaneously within a few weeks.

**Table 3:** Treatment toxicity

**Hematological toxicity**	
*Leukocytopenia*	26 (96%)
Grade 3	6 (22%)
Grade 4	17 (63%)
*Thrombocytopenia*	18 (67%)
Grade 3	3 (11%)
Grade 4	6 (22%)
*Anemia*	26 (96%)
Grade 3	3 (11%)
Grade 4	0 (0%)
*Elevated creatinine*	11 (41%)
Grade 3	0
Grade 4	0
*Skin toxicity*	6 (22%)
Grade 3	0
Grade 4	0
**Other toxicity**	16 (59%)
*Postoperative complications*	
Acute kidney failure	1
Cerebellitis	1
Anastomotic leakage	1
Burst abdomen	1
*Chemotherapy and radiotherapy*	
Nausea	7
Neutropenic fever	3
Gastritis	3
Diarrhea	1
Constipation	1
Urinary tract infection	1
Pulmonary embolism	1
Hyperkalemia	1

### Efficacy

#### Abdominal recurrence-free survival

**Fig. 3:**
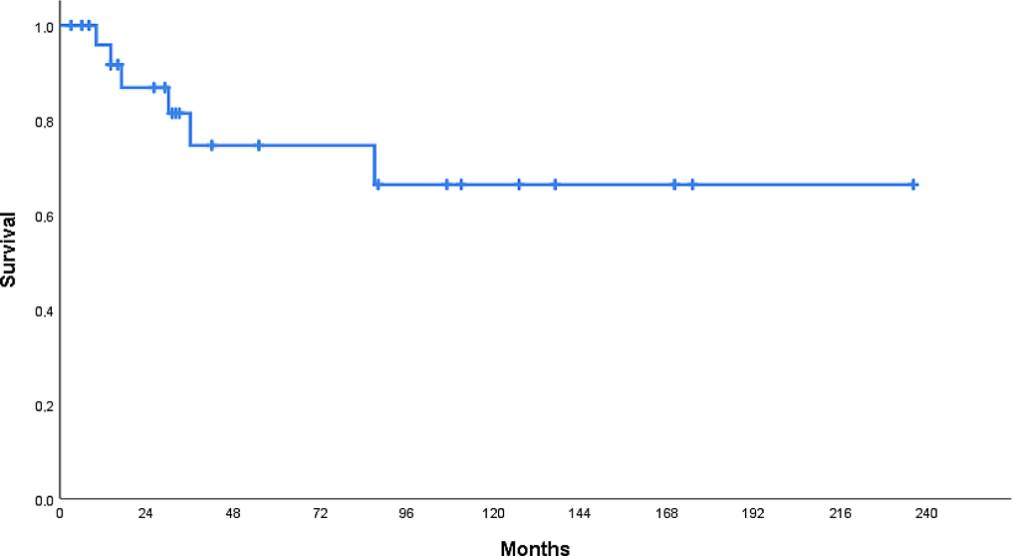
Abdominal recurrence-free survival in months

The rate of abdominal recurrence-free survival was 95.8% (±4.1%) at 1‑year follow-up, 74.6% (±10.1%) at 3 years, 74.6% (±10.1%) at 5 years, and 66.3% (±11.9%) at 10 years (see Fig. 3).

Tumors <10 cm vs. ≥10 cm in diameter showed differences in the length of ARFS, yet these were not significant (*p* = 0.076, univariate analysis). ARFS rates were slightly better in G2 tumors compared to G3 tumors, in patients who received more than six hyperthermia treatment sessions, and in younger patients. Still, the differences were not significant (see Table 4). In multivariate Cox regression analysis, none of the variables were found to be an independent prognostic factor but no conclusions should be drawn from this finding in view of the low number of events. In case of recurrence (*n* = 15), the decision to perform chemotherapy (*n* = 4), repeat chemoradiotherapy (*n* = 1), radiotherapy (*n* = 1), surgical resection (*n* = 5), or palliative treatment (*n* = 4) was made on a case-by-case basis. Local recurrence appeared infield in 3 patients and outfield in 3 patients. Regarding the 24 patients who received the combination of doxorubicin and ifosfamide, no differences in ARFS could be observed with and without the use of hyperthermia.

**Table 4:** Outcome and prognostic factors

5‑year survival					
	*n*	ARFS	DMFS	DFS	OS
*Sex*					
Male	14	66.6% *p* = 0.39	60.1% *p* = 0.91	36.4% *p* = 0.61	48.8% *p* = 0.32
Female	13	85.7%	75.5%	62.9%	72.9%
*Age*					
<60 years	11	87.5% *p* = 0.12	51.9% *p* = 0.13	41.6% *p* = 0.67	53.0% *p* = 0.76
≥60 years	16	64.3%	80.0%	54.8%	66.2%
*Histology*					
Liposarcoma	13	75.8% *p* = 0.91	58.6% *p* = 0.52	39.9% *p* = 0.57	57.5% *p* = 0.78
Other sarcoma	14	72.7%	77.4%	58.0%	62.3%
*Tumor diameter*					
<100 mm	13	85.7% *p* = 0.08	56.3% *p* = 0.46	43.8% *p* = 0.67	61.9% *p* = 0.50
≥100 mm	14	64.5%	78.6%	52.4%	58.6%
*Primary vs. recurrent*					
Primary	18	72.2% *p* = 0.32	73.1% *p* = 0.35	52.2% *p* = 0.87	64.5% *p* = 0.75
Recurrent	9	80.0%	55.6%	41.7%	53.3%
*Chemotherapy given*					
<2 cycles	25	50%^a^ *p* = 0.42	50%^a^ *p* = 0.92	0%^a^ *p* = 0.59	50%^a^ *p* = 0.80
≥2 cycles	2	74.8%	65.3%	48.1%	63.1%
*Radiotherapy method*					
3D	19	79.4% *p* = 0.53	60.8% *p* = 0.20	49.0% *p* = 0.56	61.1% *p* = 0.93
Intensity-modulated	8	68.6%	75.0%	28.6%	55.6%
*Treatment delay*					
<5 d	23	71.2% *p* = 0.33	77.2% *p* = 0.36	54.0% *p* = 0.55	67.7% *p* = 0.11
>5 d	4	100%	25.0%	25.0%	25.0%
*Hyperthermia*					
<5 sessions	8	85.6% *p* = 0.25	65.0% *p* = 0.67	51.7% *p* = 0.37	51.3% *p* = 0.34
≥5 sessions	7	53.6%	71.4%	38.1%	83.3%
*Grading*					
G2	12	80.8% *p* = 0.74	64.8% *p* = 0.60	55.6% *p* = 0.56	72.7% *p* = 0.25
G3	14	78.6%	67.7%	44.8%	42.3%

^a^Follow-up was not long enough

*ARFS* Abdominal recurrence-free survival, *DMFS* Distant metastasis-free survival, *DFS* Disease-free survival, *OS* Overall survival

#### Distant metastasis-free survival

Ten patients developed distant metastases, which initially occurred in the lungs in six cases, in the bone in three, and in the duodenum in one case. In 90% of cases, the metastases developed within 2 years of the start of treatment. The DMFS rate was 67.2% (±9.7%) at 3 years, 67.2% (±9.7%) at 5 years, and 59.7% (±11.1%) at 10 years. The difference in DMFS between recurrences and primary tumors was noticeable: 56% of patients with recurrent tumors developed further recurrences compared to 32% of those with primary tumors. In the case of DMFS, no independent predictors could be identified by multivariate analysis in the context of a low number of events. There was no difference in DMFS between patients with and without hyperthermia treatment.

#### Disease-free survival

Disease-free survival was 48.1% (±10.8%) at 3 years, 48.1% (±10.8%) at 5 years, and 36.1% (±11.0%) at 10 years. No confounding factors could be identified. Eleven patients received second-line therapy, consisting of surgery for recurrent disease in five cases, radiotherapy in one case, and systemic therapy in five cases. Four patients received palliative treatment.

#### Overall survival

**Fig. 4:**
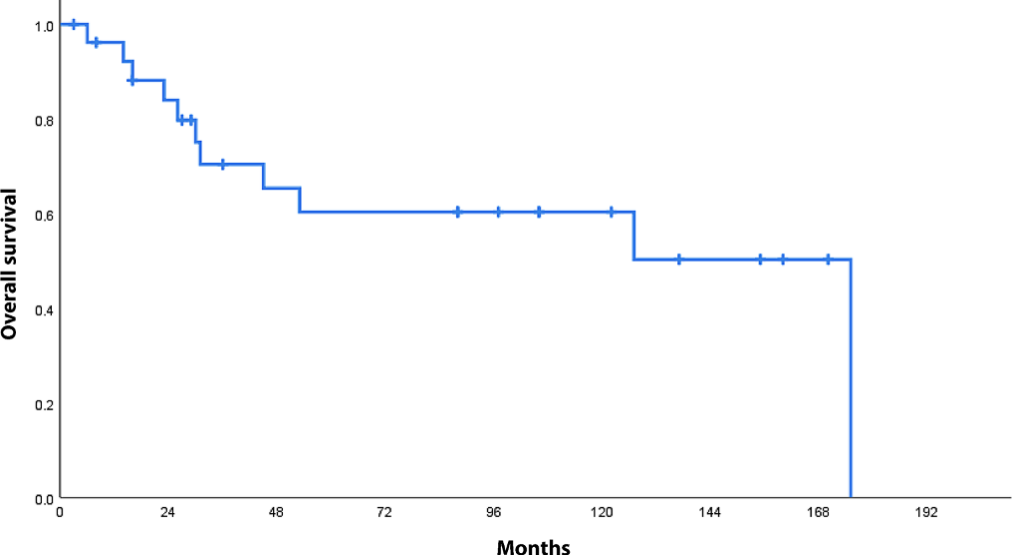
Overall survival in months

On the cut-off date for analysis, 16 patients (59%) were still alive and 2 (7%) had been lost to follow-up. Overall survival was 70.4% (±9.5%) at 3 years, 60.3% (±10.5%) at 5 years, and 60.3% (±10.5%) at 10 years (see Fig. 4). Over the long term, women and patients with G2 tumors had better overall survival, but the difference was not significant. Recurrent disease patients who were diagnosed early enough that the recurrent tumors could be treated survived for a median of 40 months.

## Discussion

Concurrent chemoradiotherapy protocols combining chemotherapy with radiation treatment have now gained acceptance as modalities for treating a wide range of locally advanced solid tumors in the neoadjuvant setting [13–15]. Retroperitoneal sarcomas pose similar challenges to surgeons regarding local resectability and recurrence rates after surgical resection alone. Nevertheless, neoadjuvant chemoradiotherapy is not a standard treatment for these tumor entities, especially after the negative results of the EORTC-STRASS study [10]. We administer CCRT to patients with high- or intermediate-grade sarcomas on a case-by-case decision basis. Our experience is based on the treatment of sarcomas of the trunk and extremities. As early as 1999, Sauer et al. demonstrated in 22 patients initially classified as inoperable that secondary resectability could be achieved with neoadjuvant chemoradiotherapy [16]. However, almost half of their patients developed high-grade toxicities, and one patient died (4%). Given the poor prognosis of sarcoma patients without resection, treatment toxicity was rated as acceptable because it achieved resectability. A later study of neoadjuvant CRT and surgery in 53 soft tissue sarcoma patients by Stubbe et al. resulted in a high long-term local recurrence rate of 90% [11] and one postoperative death (2%). Fourteen patients in their population had retroperitoneal sarcoma. The toxicity of treatment of RPS patients was not higher than that of patients with soft tissue sarcoma of the extremities. Therefore, the concept was further established in the following years. We now report the results of the treatment of 27 cases of retroperitoneal and intra-abdominal sarcoma. This constitutes the most extensive published series of RPS patients treated with neoadjuvant concurrent chemoradiotherapy.

Our analysis of the data shows that the side effects of treatment are manageable, even when the combination of radiotherapy and chemotherapy is administered in a single-center setting directed by one department. Our treatment concept includes intensive supportive therapy with parenteral nutrition and antiemetic therapy as needed as well as early stimulation therapy in case of hematological toxicity. The findings of the RTOG-9514 study highlight the importance of supportive treatment [17]. Five percent of deaths observed in RTOG-9514 occurred in patients treated using a multicenter and multimodal approach. According to our analysis, hematological and gastrointestinal toxicities were the most critical factors, but they were well controlled by parenteral nutrition, and no increase in postoperative morbidity occurred.

Analysis of our population showed that neoadjuvant CCRT can achieve excellent local control of both surgically resected and inoperable RPS. Regarding the EORTC-STRASS study [10], the 5‑year local control rate was 75% in the present analysis compared to only 47.6% in the EORTC-STRASS study. Moreover, our patient population had a less favorable prognosis as it was composed almost entirely of patients with high- or intermediate-grade tumors, whereas 34.6% of patients included in the STRASS study had low-grade sarcomas. Nevertheless, the benefit of neoadjuvant concurrent chemoradiotherapy must be evaluated in conjunction with the results of other studies. A systematic and prospective analysis of the data is favorable, especially in high-grade sarcomas. For example, Gronchi et al. [18, 19] achieved a local control rate of 63% with a neoadjuvant CCRT regimen consisting of radiotherapy up to a total dose of 50.4 Gy and high-dose ifosfamide chemotherapy. Their treatment protocol was also feasible. They also included patients with low-grade RPS but required a minimum tumor size of 5 cm in diameter. The latter size specification was not always met in our study. Studies of neoadjuvant chemotherapy alone are also rare [20].

Encouraged by the findings of Issels et al. [21], we administered hyperthermia treatment in addition to chemotherapy in almost half of our patients. In the end, we could not detect any significant difference between the patients who received hyperthermia and those who did not. This may be due to several factors. The main reason might be the small size of the sample, which resulted in reduced statistical power. Furthermore, it cannot be excluded that chemoradiotherapy alone might have already had a sufficient local therapeutic effect. Finally, it can be concluded that hyperthermia did not result in increased toxicity, so adding regional hyperthermia to the regimen as described by Issels et al. [22] is not problematic. Neoadjuvant concurrent chemoradiotherapy can be administered on a case-by-case basis and proved itself feasible and effective regarding local control rates, independent of their histological subtype.

## Conclusion

The neoadjuvant concurrent chemoradiotherapy regimen established for selected patients with retroperitoneal sarcoma is feasible and does not increase postoperative morbidity. In cases with surgical tumor resection, this approach achieved excellent local control rates, better than those obtained with radiotherapy alone. This constitutes an important signal that combined chemoradiation might be superior to radiotherapy alone in retroperitoneal sarcomas and should be prospectively evaluated in future analyses. The rate of distant metastasis of high- and intermediate-grade sarcomas remains high and needs further improvement.

## References

[1] American Cancer Society (2017) Cancer facts and figures 2017. https://www.cancer.org/research/cancer-facts-statistics/all-cancer-facts-figures/cancer-facts-figures-2017.html. Accessed 25 Sept 2020

[2] American Cancer Society (2019) Cancer facts and figures 2019. https://www.cancer.org/research/cancer-facts-statistics/all-cancer-facts-figures/cancer-facts-figures-2019.html. Accessed 25 Sept 2020

[3] Schütte J, Bauer S, Brodowicz T et al (2019) German guidelines for soft tissue sarcoma. https://www.onkopedia.com/de/onkopedia/guidelines/weichgewebssarkome-maligne-weichgewebstumoren-des-erwachsenen/@@guideline/html/index.html. Accessed 25 Sept 2020

[4] Gronchi A, Strauss DC, Miceli R (2016). Variability in patterns of recurrence after resection of primary retroperitoneal sarcoma (RPS): a report on 1007 patients from the multi-institutional collaborative RPS working group. Ann Surg.

[5] Jakob J, Lesluyes T, Simeonova-Chergou A (2020). Impact of preoperative treatment on the CINSARC prognostic signature: translational research results from a phase 1 trial of the German Interdisciplinary Sarcoma Group (GISG 03). Strahlenther Onkol.

[6] Kirste S, Landenberger N, Scholber J (2019). Retroperitoneal soft tissue sarcoma: low-dose neoadjuvant radiation therapy followed by surgery with or without intraoperative radiotherapy and adjuvant radiation therapy. Strahlenther Onkol.

[7] Dunst J (2016). Prä- oder postoperative Strahlentherapie bei retroperitonealen Sarkomen unverzichtbar. Strahlenther Onkol.

[8] Gilbeau L, Kantor G, Stoeckle E (2002). Surgical resection and radiotherapy for primary retroperitoneal soft tissue sarcoma. Radiother Oncol.

[9] O’Sullivan B, Davis AM, Turcotte R (2002). Preoperative versus postoperative radiotherapy in soft-tissue sarcoma of the limbs: a randomised trial. Lancet.

[10] Bonvalot S, Gronchi A, Le Péchoux C, Swallow CJ, Strauss D, Meeus P, van Coevorden F, Stoldt S, Stoeckle E, Rutkowski P, Rastrelli M, Raut CP, Hompes D, De Paoli A, Sangalli C, Honoré C, Chung P, Miah A, Blay JY, Fiore M, Stelmes JJ, Dei Tos AP, Baldini EH, Litière S, Marreaud S, Gelderblom H, Haas RL (2020). Preoperative radiotherapy plus surgery versus surgery alone for patients with primary retroperitoneal sarcoma (EORTC-62092: STRASS): a multicentre, open-label, randomised, phase 3 trial. Lancet Oncol.

[11] Stubbe F, Agaimy A, Ott O (2016). Effective local control of advanced soft tissue sarcoma with neoadjuvant chemoradiotherapy and surgery: a single institutional experience. Cancer Radiother.

[12] Department of Health (2009) Common terminology criteria for adverse events 4.0. https://www.eortc.be/services/doc/ctc/CTCAE_4.03_2010-06-14_QuickReference_5x7.pdf. Accessed 25 Sept 2020

[13] Sauer R, Becker H, Hohenberger W (2004). Preoperative versus postoperative chemoradiotherapy for rectal cancer. N Engl J Med.

[14] Sauer R, Liersch T, Merkel S (2012). Preoperative versus postoperative chemoradiotherapy for locally advanced rectal cancer: results of the German CAO/ARO/AIO-94 randomized phase III trial after a median follow-up of 11 years. J Clin Oncol.

[15] Shapiro J, van Lanschot JJB, Hulshof MCCM (2015). Neoadjuvant chemoradiotherapy plus surgery versus surgery alone for oesophageal or junctional cancer (CROSS): long-term results of a randomised controlled trial. Lancet Oncol.

[16] Sauer R, Schuchardt U, Hohenberger W, Wittekind C, Papadopoulos T, Grabenbauer GG, Fietkau R (1999). Neoadjuvant radiochemotherapy in soft tissue sarcomas. Optimization of local functional tumor control. Strahlenther Onkol.

[17] Kraybill WG, Harris J, Spiro IJ (2006). Phase II study of neoadjuvant chemotherapy and radiation therapy in the management of high-risk, high-grade, soft tissue sarcomas of the extremities and body wall: Radiation Therapy Oncology Group trial 9514. J Clin Oncol.

[18] Gronchi A, De Paoli A, Dani C (2014). Preoperative chemo-radiation therapy for localised retroperitoneal sarcoma: a phase I–II study from the Italian Sarcoma Group. Eur J Cancer.

[19] De Sanctis R, Giordano L, Colombo C, De Paoli A, Navarria P, Sangalli C, Buonadonna A, Sanfilippo R, Bertola G, Fiore M, Marrari A, Navarria F, Bertuzzi A, Casali PG, Basso S, Santoro A, Quagliuolo V, Gronchi A (2017). Long-term follow-up and post-relapse outcome of patients with localized retroperitoneal sarcoma treated in the Italian Sarcoma Group-Soft Tissue Sarcoma (ISG-STS) protocol 0303. Ann Surg Oncol.

[20] Gronchi A, Palmerini E, Quagliuolo V, Martin Broto J, Lopez Pousa A, Grignani G, Brunello A, Blay JY, Tendero O, Diaz Beveridge R, Ferraresi V, Lugowska I, Merlo DF, Fontana V, Marchesi E, Braglia L, Donati DM, Palassini E, Bianchi G, Marrari A, Morosi C, Stacchiotti S, Bagué S, Coindre JM, Dei Tos AP, Picci P, Bruzzi P, Casali PG (2020). Neoadjuvant chemotherapy in high-risk soft tissue sarcomas: final results of a randomized trial from Italian (ISG), Spanish (GEIS), French (FSG), and Polish (PSG) sarcoma groups. J Clin Oncol.

[21] Issels RD, Lindner LH, Verweij J (2010). Neo-adjuvant chemotherapy alone or with regional hyperthermia for localised high-risk soft-tissue sarcoma: a randomised phase 3 multicentre study. Lancet Oncol.

[22] Issels RD, Lindner LH, Verweij J (2018). Effect of neoadjuvant chemotherapy plus regional hyperthermia on long-term outcomes among patients with localized high-risk soft tissue sarcoma: the EORTC 62961-ESHO 95 randomized clinical trial. JAMA Oncol.

